# What Is Going On Around Here? Intolerance of Uncertainty Predicts Threat Generalization

**DOI:** 10.1371/journal.pone.0154494

**Published:** 2016-05-11

**Authors:** Jayne Morriss, Birthe Macdonald, Carien M. van Reekum

**Affiliations:** Centre for Integrative Neuroscience and Neurodynamics, School of Psychology and Clinical Language Sciences, University of Reading, Reading, United Kingdom; Swansea University, UNITED KINGDOM

## Abstract

Attending to stimuli that share perceptual similarity to learned threats is an adaptive strategy. However, prolonged threat generalization to cues signalling safety is considered a core feature of pathological anxiety. One potential factor that may sustain over-generalization is sensitivity to future threat uncertainty. To assess the extent to which Intolerance of Uncertainty (IU) predicts threat generalization, we recorded skin conductance in 54 healthy participants during an associative learning paradigm, where threat and safety cues varied in perceptual similarity. Lower IU was associated with stronger discrimination between threat and safety cues during acquisition and extinction. Higher IU, however, was associated with generalized responding to threat and safety cues during acquisition, and delayed discrimination between threat and safety cues during extinction. These results were specific to IU, over and above other measures of anxious disposition. These findings highlight: (1) a critical role of uncertainty-based mechanisms in threat generalization, and (2) IU as a potential risk factor for anxiety disorder development.

## Introduction

Fear learning is an adaptive process, by which an organism can associate neutral cues (conditioned stimulus, e.g. a visual stimulus such as a shape) with aversive outcomes (unconditioned stimulus, e.g. shock, loud tone). Repeated presentations of a neutral cue with an aversive outcome can result in responding to the neutral cue alone (conditioned response). This learned association can also be extinguished by repeatedly presenting the threat cue without the aversive outcome, a process known as fear extinction [1]. Recent research suggests that threat can be generalized to cues that share conceptual or perceptual similarity during fear acquisition [2–7]. This effect has been shown predominately with gradients or slopes of responding, where physiological indices such as skin conductance or startle responses are found to vary parametrically with similarity to the threat cue [2, 4, 8].

Studies examining the neural underpinnings of threat generalization have shown the amygdala and insula to respond to the potential threat value of cues, with parametrically greater responses in these regions to cues most similar to the threat cue. The ventromedial prefrontal cortex, a region known to be crucially involved in fear extinction [9, 10], has been shown to respond to the safety value of cues, with parametrically greater responses in the ventromedial prefrontal cortex for cues most dissimilar to the threat cue [3, 11]. Notably, flatter gradients of discrimination, suggesting greater threat generalization, are found for patients with post-traumatic stress disorder (PTSD) [12], panic disorder [13] and generalized anxiety disorder [14, 15].

Currently, it is still under debate whether threat generalization behavior exists before disorder onset as part of a dispositional tendency for anxiety. Previous work has focused on examining the link between the extent of threat generalization and the Spielberger State-Trait Anxiety Inventory (STAI) [16], which is a measure of dispositional fear and anxiety responsiveness. However, this approach has resulted in mixed findings [6, 8, 11]. For example, Dunsmoor et al. (2011) found trait anxiety to predict increased functional connectivity between the amygdala and sensory regions for the safe cue that was most perceptually similar to the threat cue. Torrents-Rodas et al. (2013) found no evidence of greater threat generalization in eyeblink startle, skin conductance or ratings for high trait anxious individuals. The equivocal results between trait anxiety and threat generalization may stem from a lack of construct specificity within the STAI measure, which does not capture any specific elicitors of fear or anxiety.

One potential factor that may exacerbate threat generalization is Intolerance of Uncertainty (IU). IU can be defined as a dispositional tendency that affects how uncertain situations are perceived and interpreted. Individuals with high IU scores tend to find uncertain situations inherently aversive and anxiety provoking. During experienced threat uncertainty, high IU individuals may be prone to generalizing threat to ambiguous, neutral, or even positive cues [17]. Originally, IU was considered to be specifically related to Generalized Anxiety Disorder [17]. However, growing evidence suggests that IU may be a transdiagnostic factor across many anxiety and mood disorders [18–21]. Furthermore, the development of new disorder-specific IU scales [22] suggests that IU may be applicable to anxiety disorders that are associated with compromised fear extinction, such as specific phobia, PTSD and panic disorder.

Recent research has begun to assess the role of IU in fear acquisition [23], extinction [24] and generalization [25]. For example, during simple conditioning, where cues vary in color, such that a blue square represents the threat cue and a yellow square represents the safe cue, individuals scoring high on IU have been shown to generalize threat to safety cues and to continue responding to threat cues during extinction [24]. The extent to which future threat uncertainty sensitivity is associated with overestimating the threat value of safety cues can be further examined by embedding cues that vary parametrically on a specific dimension, such as color, shape, or size. Given the paucity of literature on IU and mixed results with trait anxiety above, it seems pertinent to examine whether IU predicts threat generalization behavior. Understanding associations between IU and threat generalization could help characterize IU-based maintenance of anxiety, with implications for targeted treatment [23, 26, 27]. For example, recent research suggests an important role of IU upon avoidance behaviors during safe contexts for panic disorder patients [19, 28, 29].

Typically in fear generalization designs, the conditioned response is assessed by presenting CS+ and CS- stimuli before the introduction of generalization stimuli [11, 30]. Here we embedded perceptually graded threat and safety cues during acquisition and extinction, in order to assess how generalization changes across these different phases. With this design, we assessed the relationship between individual differences in self-reported IU and in psychophysiological correlates of threat generalization during acquisition and extinction. We measured skin conductance response (SCR) and uneasiness ratings whilst participants performed the conditioning task. We used an aversive sound as an unconditioned stimulus and visual shapes that varied in size parametrically as conditioned stimuli, similar to previous conditioning research [3, 4, 10, 31, 32]. In general, we hypothesized that, during fear acquisition and extinction, responding would be graded parametrically depending on similarity between the threat and safety cues, and that this effect would be larger in acquisition, compared to extinction. Furthermore, we hypothesized that sensitivity to future threat uncertainty as measured by IU would predict the extent of responding to parametrically graded threat and safety cues during acquisition and extinction. For SCR and uneasiness ratings, we expected to observe high IU to be associated with greater generalization across cues during acquisition, and continued responding to cues during extinction relative to low IU, who would show less generalization during acquisition and a greater reduction in responding to threat cues during extinction. We tested the specificity of the associations between threat generalization and IU by comparing with broader measures of anxiety, such as Spielberger State-Trait Anxiety Inventory, Trait Version (STAIX-2) [16] and Penn State Worry Questionnaire (PSWQ) [33].

## Method

### Participants

54 volunteers took part in this study (mean age = 19.48; 49 females & 5 males). The sex distribution of the participants reflected typical sampling from the recruitment site (Psychology undergraduate students). All participants had normal or corrected to normal vision. Participants provided written informed consent and received course credit for their participation. The procedure was approved by the University of Reading’s Research Ethics Committee.

### Procedure

Participants arrived at the laboratory and were informed on the procedures of the experiment. Firstly, participants were taken to the testing booth and given a consent form to sign as an agreement to take part in the study. Secondly, to assess emotional disposition, we asked participants to complete a series of questionnaires presented on a computer in the testing booth. Thirdly, participants completed a short perceptual task (please see Text A in S1 File). Next, physiological sensors were attached to the participants’ non-dominant hand. Participants were simply told they would see visual stimuli (squares) and hear loud sounds, which they may find aversive. Participants were presented with the conditioning task on a computer, whilst skin conductance, interbeat interval and behavioral ratings were recorded. After the task, subjects were asked to rate the valence and arousal of the sound stimulus using Likert scales that were 1 (Valence: very negative; Arousal: calm) to 9 (Valence: very positive; Arousal: excited).

### Conditioning task

The conditioning task was designed using E-Prime 2.0 software (Psychology Software Tools Ltd, Pittsburgh, PA). Visual stimuli were presented on a computer screen, which displayed stimuli at 60 Hz on a 600 x 800 pixel screen. Participants sat approx. 60 cm from the computer screen. Sound stimuli were presented through headphones.

Visual stimuli were four yellow squares that varied in size (visual angle of smallest to largest square: 2.18° x 3.67°; 2.75° x 4.63°; 3.28° x 5.59°; 3.85° x 6.55°). The aversive sound stimulus consisted of a fear inducing female scream (sound number 277) from the International Affective Digitized Sound battery (IADS-2) and which has been normatively rated as unpleasant (M = 1.63, SD = 1.13) and arousing (M = 7.79, SD = 1.13) (Bradley & Lang, 2007). We used Audacity 2.0.3 software (http://audacity.sourceforge.net/) to shorten the female scream to 1000 ms in length and to amplify the sound by 15 db, resulting in a 90 db sound (~5 db error in the range of measurement). The volume of the sound was standardized across the experiment by using fixed volume settings on the presentation computer and verified by an audiometer prior to each session. The experiment was run in the same lab.

**Fig 1:**
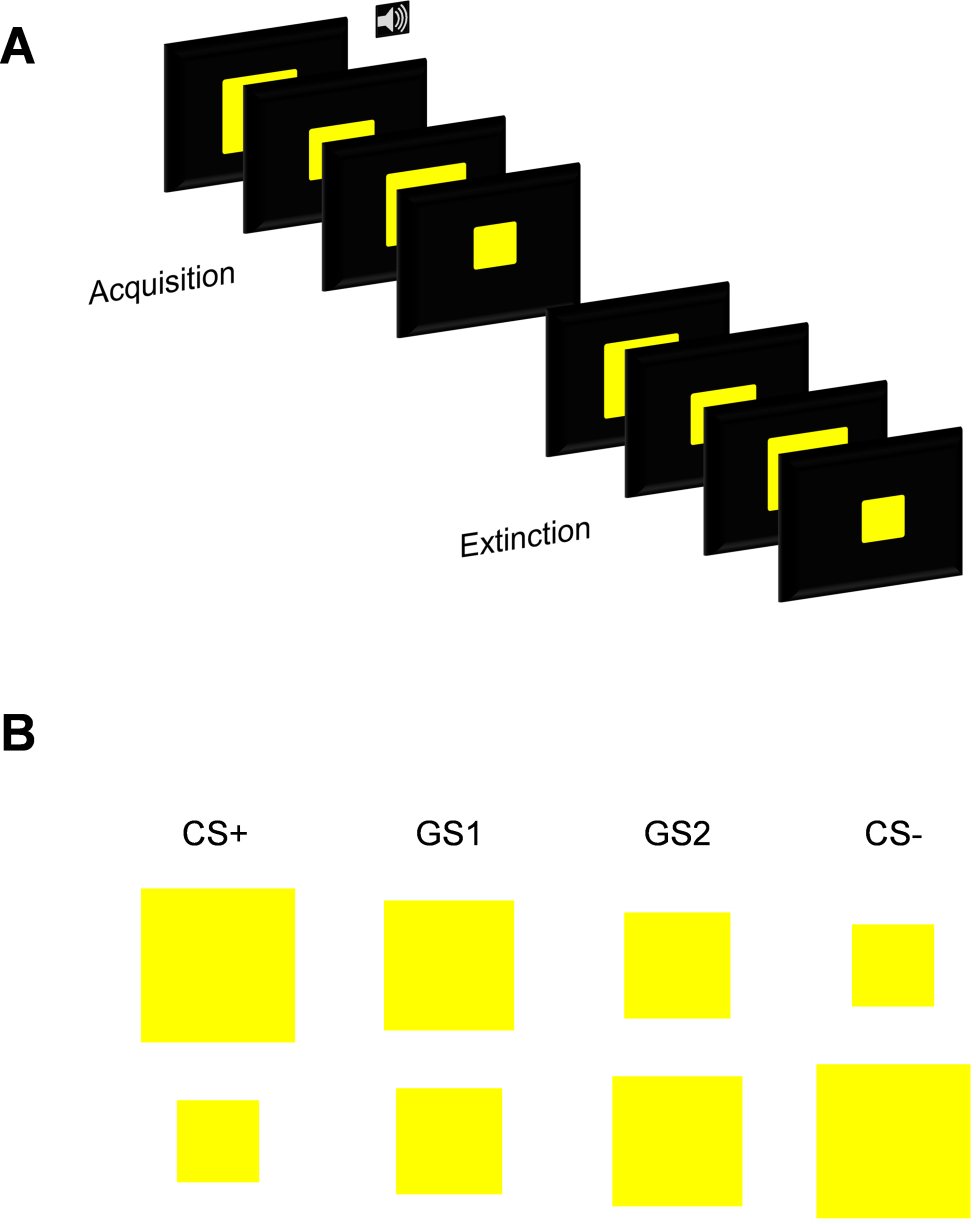
Experimental design. Illustration depicting: (A) Example trial order from the acquisition and extinction phases. (B) Example of stimulus sizes and assignment of stimulus counterbalancing.

The conditioning task comprised of two learning phases: acquisition and extinction. Both learning phases consisted of 4 blocks each (see Fig 1). The acquisition phase consisted of 36 trials (12 CS+ (6 paired and 6 unpaired), 8 GS1, 8 GS2, 8 GS3) and the extinction phase 40 trials (10 CS+, 10 GS1, 10 GS2, 10 GS3). Experimental trials within the conditioning task were pseudo-randomized into an order, which resulted in no more than three presentations of the same stimulus in a row.

In acquisition, either the largest or smallest square was paired with an aversive 90 db scream (CS+) at a 50% reinforcement schedule, whilst the other three squares were presented alone (GS1, GS2, GS3). In extinction, all the stimuli were presented alone (CS+, GS1, GS2, GS3). Visual stimuli were presented for a total of 1500 ms, and the auditory stimulus (US) lasted for 1000 ms. Reinforced CS+ included the US, played 500 ms after onset of the CS+ and co-terminating with it. A jittered ITI, ranging between 4700 ms and 6500 ms, followed each stimulus presentation. Participants were instructed to attend and listen to the stimulus presentations, as well as respond to a rating scale that followed each block of trials. The rating scale presented the participants with each stimulus depicted above it (in the same size) and asked how 'uneasy' the participant felt when viewing the stimulus, where the scale was 1 'not at all'- 9 'extremely'. Participants used the number keys on the keyboard with their dominant hand to respond.

Conditioning contingencies were counterbalanced, with half of the participants receiving the aversive scream with the smallest square and the other half of participants receiving the aversive scream with the largest yellow square.

### Questionnaire task

To assess emotional disposition, we administered the Spielberger State-Trait Anxiety Inventory, Trait Version (STAIX-2) [16], Penn State Worry Questionnaire (PSWQ) [33], and Intolerance of Uncertainty (IU) [34]. Similar distributions and internal reliability of scores were found between the anxiety measures, STAIX-2 (*M* = 42.88; *SD* = 9.30; range = 22–68; α = .906.), PSWQ (*M* = 48.16; *SD* = 12.19; range = 23–71; *α* = .927.) and IU (*M* = 60.77; *SD* = 16.69; range = 30–99; *α* = .918). Furthermore, anxiety measures were strongly correlated: IU with STAIX-2, *r*(41) = .531, *p* < .001, IU with PSWQ, *r*(41) = .538, *p* < .001, and STAIX-2 with PSWQ, *r*(41) = .742, *p* < .001.

### Behavioral data scoring

Rating data were reduced for each subject by calculating their average responses for each experimental condition in each phase using the E-Data Aid tool in E-Prime (Psychology Software Tools Ltd, Pittsburgh, PA). 0.02% of rating trials were missing.

### Physiological acquisition and scoring

Physiological recordings were obtained using AD Instruments (AD Instruments Ltd, Chalgrove, Oxfordshire) hardware and software. An ML138 Bio Amp connected to an ML870 PowerLab Unit Model 8/30 amplified the skin conductance and IBI signals, which were digitized through a 16-bit A/D converter at 1000 Hz. Electrodermal activity was measured with dry MLT116F silver/silver chloride bipolar finger electrodes that were attached to the distal phalanges of the index and middle fingers of the non-dominant hand. A low constant-voltage AC excitation of 22mV_rms_ at 75 Hz was passed through the electrodes, which were connected to a ML116 GSR Amp, and converted to DC before being digitized and stored. IBI was measured using a MLT1010 Electric Pulse Transducer, which was connected to the participant’s distal phalange of the ring finger of the non-dominant hand.

CS+ trials paired with the US were discarded from the analysis. All other trials were included, i.e. CS+ unpaired, GS1, GS2 and GS3. Skin conductance responses (SCR) were scored when there was an increase of skin conductance level exceeding 0.03 microSiemens. The amplitude of each response was scored as the difference between the onset and the maximum deflection prior to the signal flattening out or decreasing. SCR onsets had to be within 0.5–4 seconds following each CS onset to be included. Trials with no SCRs were scored as zero (percentage of trials scored as zero during: Acquisition, 58.9%; Extinction, 63.7%). SCR magnitudes were square root transformed to reduce skewness and were z-scored to control for interindividual differences in skin conductance responsiveness. The first trial of acquisition was excluded to reduce contamination from the orienting responses typically seen at the start of a session. IBI signal was not analysed. Trials with motion artefacts were discarded from the analysis. Motion artefacts were identified by observing distortions in both electrodermal and IBI signal during a given trial. Only 0.003% (15 from 3800) of trials were discarded. SCR magnitudes were calculated from remaining trials by averaging SCR values and zeros for each condition.

To assess whether individuals learned to discriminate between the stimuli that predicted threat of safety, two difference score metrics were calculated, one by subtracting GS3 SCR magnitude from CS+ unpaired SCR magnitude from the acquisition phase and one based on the same difference score stemming from the extinction phase. In acquisition there were 24 discriminators and 19 non-discriminators (Please see Text B in S1 File).

We conducted analyses on a total of 43 participants. Eleven subjects were excluded from these analyses (4 subjects due to computer error, 6 subjects who did not display differential responding in either acquisition or extinction, and 1 subject due to extreme SCR magnitude values during extinction which were 3 standard deviations away from the mean).

### Rating and SCR magnitude analysis

The analysis was conducted using the mixed procedure in SPSS 21.0 (SPSS, Inc; Chicago, Illinois). We conducted separate multilevel models on behavioral ratings and SCR magnitude by entering Stimulus (CS+, GS1, GS2, GS3) and Phase (Acquisition, Extinction) at level 1 and individual subjects at level 2, with IU, PSWQ and STAIX-2 entered as individual difference predictor variables. We used a diagonal covariance matrix for level 1. Random effects included a random intercept for each individual subject, where a variance components covariance structure was used. Fixed effects included Stimulus and Phase. We used a maximum likelihood estimator (for alternative analysis, please see Text C in S1 File). We corrected for multiple comparisons using the Benjamin-Hochberg False Discovery Rate procedure. For the behavioral ratings, pairwise comparisons were considered significant if *p* < .048. For the SCR analysis, pairwise comparisons were considered significant if *p* < .019.

We report the specificity of IU with respect to PSWQ and STAIX-2 where a significant interaction of IU with Stimulus or Phase x Stimulus was observed. Then, we performed follow-up pairwise comparisons on the estimated marginal means, adjusted for the predictor variables (IU, PSWQ, STAIX-2). Any interaction with IU was followed up with pairwise comparisons of the means between the conditions for IU estimated at the specific values of + or—1 SD of mean IU. These data are estimated from the multilevel model of the entire sample, not unlike performing a simple slopes analysis in a multiple regression analysis.

## Results

### Ratings

All subjects rated the sound stimulus as aversive (*M* = 2.05 *SD* = 1.07, range 1–9, where 1 = very negative and 9 = very positive) and arousing (*M* = 7.21, *SD* = 1.63, range 1–9 where 1 = calm and 9 = excited).

**Fig 2:**
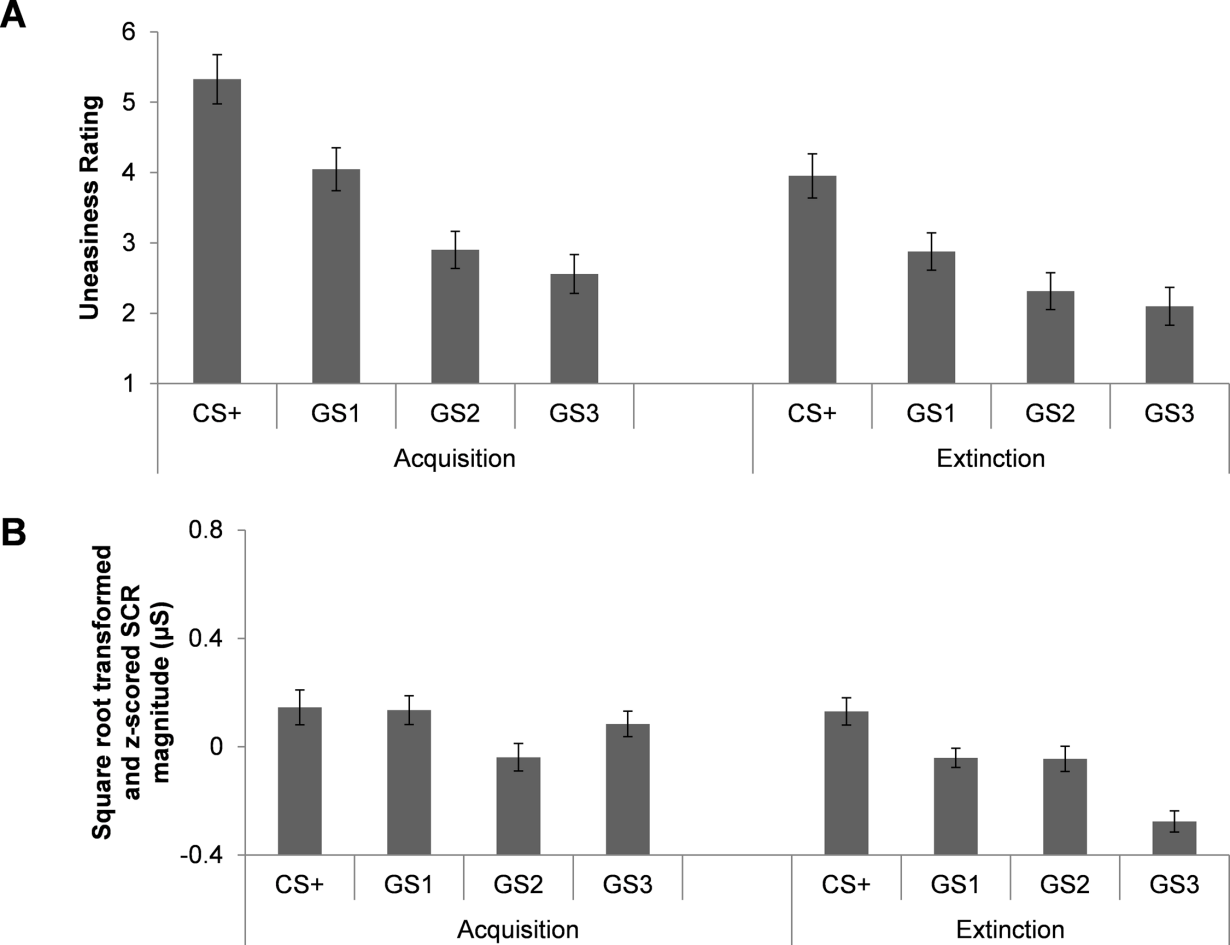
Threat generalization of ratings. Bar graphs demonstrating group ratings (A) and SCR magnitude (B) during acquisition and extinction. Bars represent standard error. Square root transformed and z-scored SCR magnitude (μS), skin conductance magnitude measured in microSiemens. Uneasiness rating, 1 = not at all and 9 = extremely.

For the uneasiness ratings, significant main effects of Stimulus, *F*(3, 168.02) = 77.262, *p* < .001, and Phase, *F*(1,169.44) = 74.104, *p* < .001 as well as an interaction between Stimulus and Phase emerged, *F*(3, 168.02) = 4.786, *p* = .004 (for descriptive statistics of ratings see Table 1 and Fig 2A, and for the estimated coefficients from the model see Table 2). As expected, participants reported feeling the greatest uneasiness to the CS+, followed by the GS1, GS2 and GS3. This perceptually graded rating of uneasiness was found for both acquisition and extinction *p*’s < .01. Furthermore, participants rated greater uneasiness for each stimulus during the acquisition phase, compared to the extinction phase, *p*’s < .01.

**Table 1:** Summary of means (SD) for each dependent measure as a function of condition during the acquisition and extinction phase.

	Acquisition				Extinction			
Measure	CS+	GS1	GS2	GS3	CS+	GS1	GS2	GS3
Uneasiness rating	5.32 (2.34)	4.05 (2.19)	2.90 (1.92)	2.56 (1.68)	3.95 (2.88)	2.88 (1.94)	2.31 (1.68)	2.10 (1.52)
Square root transformed and z-scored SCR magnitude (μS)	0.15 (0.48)	0.14 (0.36)	-0.03 (0.36)	0.08 (0.34)	0.13 (0.36)	-0.04 (0.26)	-0.04 (0.33)	-0.28 (0.26)

Note: Uneasiness rating, 1 = not at all and 9 = extremely; SCR magnitude (μS), square root transformed and z-scored skin conductance magnitude measured in microSiemens

**Table 2:** Multilevel model predicting uneasiness ratings and SCR magnitude from Stimulus, Phase and IU estimates.

Predictor	*df*	Estimate	*SE*	*t*
	Uneasiness rating			
Intercept	49.367	2.0988	0.2687	7.812**
Stimulus	60.890	1.8546	0.2153	8.570**
Phase	58.108	0.4592	0.1576	3.191**
Stimulus x Phase	107.834	0.9130	0.3493	2.763**
Phase x IU	57.129	-0.0062	0.0205	-0.5280
	Square root transformed and z-scored SCR magnitude (μS)			
Intercept	43.0000	-0.2762	0.0386	-7.147**
Stimulus	80.6560	0.4058	0.0634	6.399**
Phase	82.8480	0.3598	0.0609	5.907**
Stimulus x Phase	151.1940	-0.3440	0.1018	-3.380**
Stimulus x Phase x IU	151.1940	-0.0198	0.0075	-2.635**
				

Note: Uneasiness rating, 1 = not at all and 9 = extremely; SCR magnitude (μS), square root transformed and z-scored skin conductance magnitude measured in microSiemens.

* p < .05

** p < .01

There was a significant interaction between Phase x IU, *F*(1, 169.44) = 4.550, *p* = .029, such that low IU was associated with greater reduction in uneasiness ratings from acquisition (*M* = 3.78, *SD* = 2.72) to extinction (*M* = 2.60, *SD* = 2.66), whilst high IU was associated with less reduction in uneasiness ratings from acquisition (*M* = 3.63, *SD* = 2.72) to extinction (*M* = 3.02, *SD* = 2.66), *p* < .01.

No other significant effects of IU, PSWQ or STAIX-2 were found on the ratings, max *F* = 2.558, *p*’s >.05.

### SCR magnitude

A significant main effect of Stimulus, *F*(3,165.23) = 8.314, *p* < .001 was found for SCR magnitude. Over both phases, participants displayed SCR magnitudes in line with perceptual grading of the cues, CS+ vs. GS2, CS+ vs. GS3, and GS1 vs GS3, *p’s* < .01 (for descriptive statistics see Table 1 and from the estimated coefficients of the model see Table 2). However, stimuli most perceptually similar did not significantly differ, CS+ vs. GS1 and GS2 vs.GS3, *p*’s > .05. There was also a main effect of Phase, *F*(1,308.35) = 16.168 *p* < .001, such that SCR magnitude was larger during acquisition, compared to extinction, *p* < .001 (see Table 1 and Table 2). Lastly, there was a significant interaction between Stimulus and Phase, *F*(3,165.23) = 6.283, *p* < .001 (see Fig 2B). This effect was driven by a general reduction in SCR magnitude to GS1 and GS3 from acquisition to extinction, *p*’s < .01. In addition, during extinction, there was larger SCR magnitude to CS+ vs. GS1, *p* = .007, CS+ vs. GS2, *p* = .013, CS+ vs. GS3, *p* < .001, and GS1 vs. GS3, *p* < .001.

**Fig 3:**
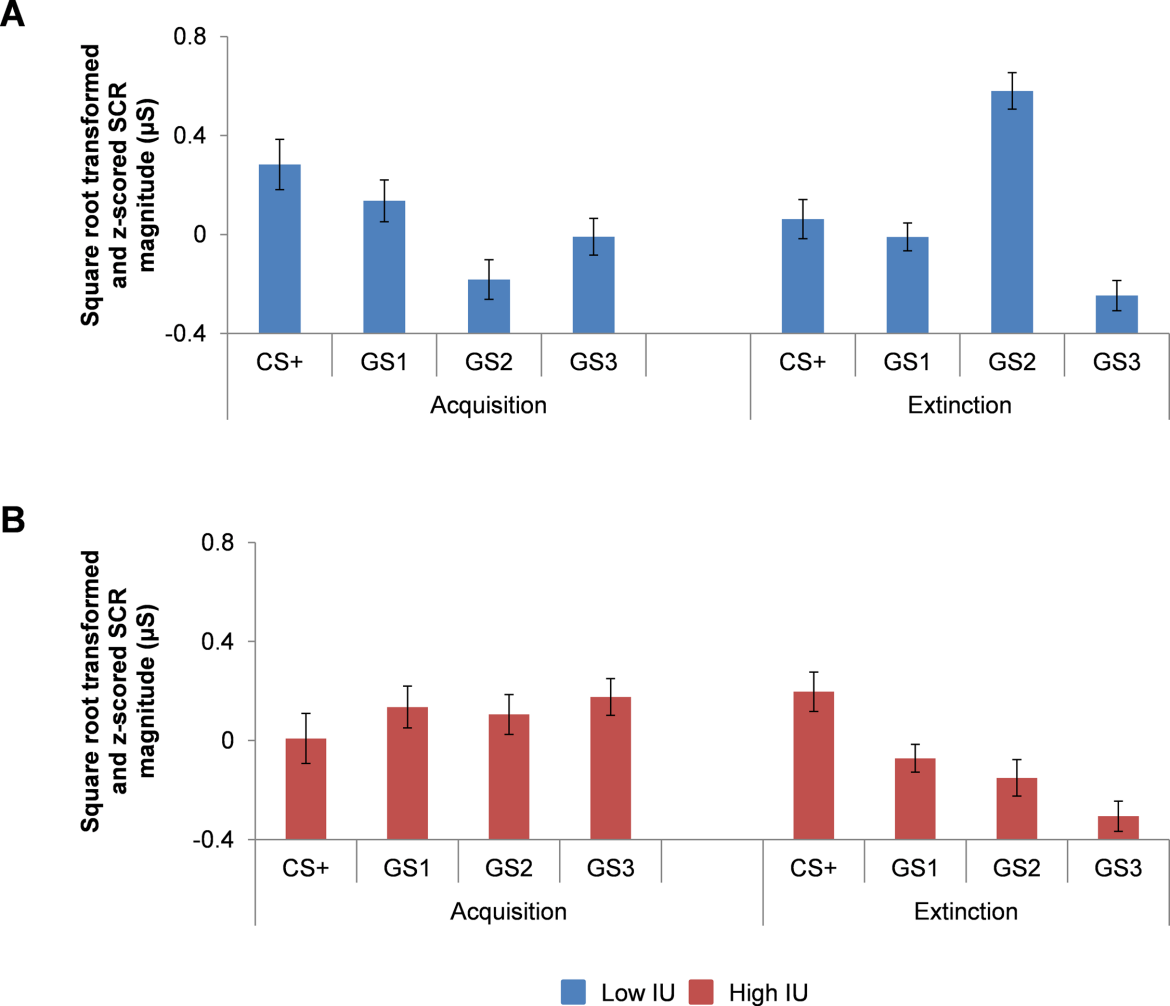
Threat generalization of skin conductance responses as a function of Intolerance of Uncertainty. Bar graphs depicting IU estimated at + or—1 SD of mean IU from the multilevel model analysis for SCR magnitude during acquisition and extinction. (A) During acquisition, lower IU was associated with larger SCR magnitudes to threat vs. safety cues, in the order of perceptual similarity to the threat cue. In the extinction phase, lower IU was associated with reduction in perceptually graded SCR magnitude discrimination to threat vs. safety cues. (B) Higher IU, however, was associated with larger SCR magnitude to all cues during acquisition, and larger SCR magnitudes to threat vs. safety cues during extinction, in the order of perceptual similarity to the threat cue. Bars represent standard error at + or – 1 SD of mean IU. Square root transformed and z-scored SCR magnitude (μS), skin conductance magnitude measured in microSiemens.

As predicted, we found a significant Stimulus x Phase x IU interaction, *F*(3,165.23) = 4.241, *p* = .006 (see Table 2 and Fig 3). Further inspection of follow-up pairwise comparisons for acquisition and extinction revealed that low IU (1 SD below the IU mean) was associated with perceptually graded SCR magnitude discrimination to threat stimuli during acquisition, with larger SCR magnitude to CS+ vs. GS2, *p* = .001, at trend for CS+ vs. GS3, *p* = .022, and for GS1 vs. GS2, *p* = .008 (see Fig 3A). In addition, low IU was associated with greater SCR magnitude to threat stimuli during extinction, with larger SCR magnitude to CS+ vs. GS3, *p* = .003, GS1 vs. GS3, *p* = .006, GS2 vs. GS3, *p* = .002. (see Fig 3A). Furthermore, low IU was associated with no differences between CS+, GS1, and GS2 stimuli during extinction, *p*’s > .4 (see Text D in S1 File and Fig A in S1 File). High IU (1 SD above the mean), however, was associated with threat generalization during acquisition, as SCR magnitudes did not significantly differ across stimuli, *p*’s >.1 (see Fig 3B). Furthermore, during extinction, high IU was associated with perceptually graded discrimination to threat stimuli, suggesting slower discrimination compared to low IU where this pattern was already observed in acquisition: CS+ vs. GS1, *p* = .007, CS+ vs. GS2, *p* = .002, CS+ vs. GS3, *p* < .001 and GS1 vs.GS3, p = .006. All other pairwise comparisons were not significant, *p*’s > .05.

A similar interaction emerged for the PSWQ measure, albeit statistically weaker than the IU measure: Phase x Stimulus x PSWQ, *F*(3,165.23) = 3.096, *p* = .028. No other significant effects of IU, PSWQ or STAIX-2 were found on SCR magnitude, max *F* = 2.238, *p*’s >.05.

## Discussion

In the current study, we show that individual differences in self-reported IU predict threat generalization during associative learning. Our data suggest that during fear acquisition, high IU is associated with greater generalization of threat to safety cues, whilst during fear extinction, high IU is associated with continuation of parametrically graded responding to threat and safety cues. The findings for IU hold when controlling for other general measures of anxious disposition such as STAI and PSWQ. These results further our understanding of previous fear conditioning studies where psychophysiological and neural patterns of responding were associated with IU during extinction [24], highlighting threat generalization as a process by which individual differences in IU may maintain or prolong extinction-resistant fear in anxiety disorders.

Using a mixed conditioning and generalization procedure, we found threat generalization during acquisition and extinction to vary as a function of cue similarity to the threat cue in SCR magnitude and ratings, in line with past work using more traditional designs [3, 4, 6–8, 11, 13, 15]. Importantly, the pattern of threat generalization in SCR magnitude during these associative learning phases differed substantially depending on individual differences in IU. During acquisition, low IU was associated with stronger discrimination between threat and safety cues. Conversely, high IU was associated with little discrimination between threat and safety cues, similar to that observed in anxiety disorder patients [13–15]. Building upon previous research examining individual differences in IU and fear extinction [23, 24], low IU predicted continued discrimination between threat and safety cues, whilst high IU predicted delayed discrimination to threat and safety cues, similar to that already observed in low IU individuals during acquisition. Crucially, these patterns of stimulus discrimination were specific to IU, over STAIX-2 and PSWQ measures. Taken together, these results suggest that high IU individuals take longer to discriminate between threat and safety cues because of threat generalization proneness. This effect may subsequently prolong the amount of exposure needed for high IU individuals to extinguish threat. The generalization of threat may serve as a key candidate marker for IU-based maintenance of fear and anxiety in disorders where generalization behavior is commonly observed, such as PTSD, specific phobia and panic disorder.

In the ratings, participants were clearly able to discriminate between threat and safety cues in a perceptually graded fashion. We observed self-reported uneasiness ratings of each phase to vary as a function of individual differences in IU. High IU was associated with less differentiation between the average uneasiness ratings for all cues during acquisition, compared to extinction. Furthermore, this result was specific to IU over PSWQ and STAIX-2. These findings suggest that IU is predictive of both ratings of uneasiness and psychophysiological responses during associative learning. The differential effects of IU on phase and cue discrimination for uneasiness ratings and psychophysiological measures may simply be due to the time between phasic cue events and the periods during which ratings were provided.

The design specifics of the current study should be further addressed in future research. Firstly, the procedure may have been quite ambiguous given the fast CS presentations, the 50% reinforcement schedule, the relatively short intertrial intervals, and mixed acquisition phase (e.g. conditioning and generalization stimuli presented together). It is possible that the effects of IU-based generalization are only found under these conditions of heightened uncertainty. However, previous studies have observed threat generalization-like behavior during simple cued extinction in high IU individuals [24]. Secondly, the generality of these findings should be tested in future studies using stimuli other than colored squares, such as faces, since classes of stimuli may inherently differ in perceptual discrimination [35]. Thirdly, using a longer extinction phase with more unpaired trials may have elucidated if individuals high in IU required more exposure to extinguish. Fourthly, this study used relatively short intertribal intervals, preventing SCR to consistently return to baseline at the end of the trial, which may have lowered the SCR rate [36]. Lastly, the sample contains mainly female participants, and future studies should more carefully balance their sample in terms of gender. All these points would benefit from further research to assess the robustness and generalizability of the findings reported here.

In conclusion, individual differences in IU predicted threat generalization, over and above other anxiety measures. Higher IU was associated with overestimating the threat value of safety cues during acquisition, which subsequently prolonged threat and safety cue discrimination during extinction. Importantly, these results highlight an opportunity for further research to explore: (1) how individual differences in IU may disrupt fear extinction processes and safety appraisals more generally, (2) how IU may be a potential predictor factor for development of anxiety disorders, and (3) further current forms of focused anxiety disorder treatment, which aim to target IU-based maintenance of fear and anxiety in disorders such as PTSD, specific phobia and panic disorder.

## Supporting Information

S1 Data Data in long format for SPSS. (XLS)

S1 File Supporting Information (DOC)

S1 Raw Data Data set for study. (XLSX)

S1 Text Syntax for MLM analysis using SPSS. (TXT)
