# Supplementary material for: What Is Going On Around Here? Intolerance of Uncertainty Predicts Threat Generalization
Source: PLoS One. 2016 May 11;11(5):e0154494. doi: 10.1371/journal.pone.0154494 (PMC4864232; doi:10.1371/journal.pone.0154494)
Supplement: S1 File — (DOC) [file pone.0154494.s002.doc]

**Text A. Perceptual discrimination task**

In order to ascertain that all participants could discriminate between the stimuli used in the main experiment, we conducted an independent perceptual discrimination task, without reinforcement.

**Task design**

Participants were presented with two diamond shapes. Participants were told to respond as quickly and accurately as possible using the keyboard as to which diamond shape was the smallest or largest, depending on the test block.

The perceptual discrimination task was designed using E-Prime 2.0 software (Psychology Software Tools Ltd, Pittsburgh, PA). Visual stimuli were presented on a computer screen, which displayed stimuli at 60 Hz on a 600 x 800 pixel screen. Participants sat approx. 60 cm from the computer screen.

Visual stimuli were four blue diamond shapes that varied in size. These shapes were simply rotated versions of the yellow squares in the conditioning task (visual angle of smallest to largest diamond: 2.18° x 3.67°; 2.75° x 4.63°; 3.28° x 5.59°; 3.85° x 6.55°).

The perceptual discrimination task comprised of two test blocks which were counterbalanced: (1) Find the smallest diamond, and (2) find the largest diamond. Each test block consisted of 24 trials, resulting in a total of 48 trials. Each trial consisted of a fixation cross presented for 1000 ms followed by two diamonds, one presented on the left and one presented on the right for 1500 ms. Participants had to respond using the keyboard (z key for left; m key for right).

Conditions comprised of the perceptual similarity between stimuli: smallest difference (e.g. 1 size away; 24 trials), medium difference (2 sizes away; 16 trials) and largest difference (3 sizes away; 8 trials).

**Behavioral data scoring**

Accuracy percentage scores were reduced for each subject by calculating their average responses for each experimental condition in each phase using the E-Data Aid tool in E-Prime (Psychology Software Tools Ltd, Pittsburgh, PA).

**Analysis**

We conducted a multilevel model on percentage accuracy scores for those subjects included in the conditioning task analysis. We entered Stimulus (smallest difference, medium difference, largest difference) at level 1 and individual subjects at level 2, with IU, PSWQ and STAIX-2 entered as covariates. We used a diagonal covariance matrix for level 1. Random effects included a random intercept for each individual subject, where a variance components covariance structure was used. Fixed effects included Stimulus. We used a maximum likelihood estimator.

**Results**

As expected, participants were generally less accurate when stimuli were the most perceptually similar, *F*(2,43.76) =48.967, *p*< .001. This effect was perceptually graded across all multiple comparisons for percentage accuracy scores; smallest difference, M = .90, SD = .08; medium difference, M = .98, SD = .07; largest difference, M = .098, SD = .07, all simple comparison *p*’s <.001, except for medium difference vs. largest difference, *p* > .05.

To check that the observed IU effects in the fear generalization task were not due to a general discrimination difficulty, we assessed IU, (and PSWQ and STAI) in relation to performance on the perceptual task without reinforcement. No anxiety measures predicted performance on the perceptual task, Max *F* = 2.245, *p* >.05. These results argue against any difficulty in perceptual discrimination as a function of IU (and PSWQ and STAI).

**Text B. Discrimination learning assessment**

Typically in classical conditioning studies, those individuals who do not display discrimination between learned threat and safety cues (a positive difference score from CS+ - CS-, or in this case GS3 rather than CS-) in the acquisition phase are excluded from the analysis of data stemming from the extinction phase . However, non-discriminators are frequently reported in anxiety disorder samples , suggesting that impaired discrimination may be an important aspect to examine. Thus, we divided subjects into groups based on a discrimination metric in acquisition. The discrimination metric was calculated by subtracting GS3 SCR magnitude from CS+ unpaired SCR magnitude in the acquisition phase. A positive difference score indicated discrimination and a negative difference score suggested non-discrimination. In acquisition there were 24 discriminators and 19 non-discriminators.

We conducted a multilevel model on SCR magnitude elicited during extinction by entering Stimulus (CS+, GS1, GS2, GS3) and Group (Acquisition discriminators [n = 24], Non-acquisition discriminators [n = 19]) at level 1 and individual subjects at level 2. We used a diagonal covariance matrix for level 1. Random effects included a random intercept for each individual subject, where a variance components covariance structure was used. Fixed effects included Stimulus and Group. We used a maximum likelihood estimator. In extinction, a main effect of Stimulus was found for SCR magnitude, *F*(3, 90.74) = 15.256, *p* < .001, but no interaction between Stimulus and Group was found for SCR magnitude, *F*(3, 90.74) = 1.363, *p* = .259. Therefore, these findings suggest that the non-acquisition discriminators still learned the CS-US contingencies, as denoted by conditioned responses to the CS+ vs. GS3 in extinction. Given these results, we only excluded subjects who displayed no differential responding to the CS+ vs. GS3 in either acquisition or extinction.

**References**

1. Phelps EA, Delgado MR, Nearing KI, LeDoux JE. Extinction learning in humans: role of the amygdala and vmPFC. Neuron. 2004;43(6):897-905.

2. LaBar KS, Gatenby JC, Gore JC, LeDoux JE, Phelps EA. Human amygdala activation during conditioned fear acquisition and extinction: a mixed-trial fMRI study. Neuron. 1998;20:937-45.

3. Lissek S, Powers AS, McClure EB, Phelps EA, Woldehawariat G, Grillon C, et al. Classical fear conditioning in the anxiety disorders: a meta-analysis. Behaviour research and therapy. 2005;43(11):1391-424.

**Text C. Alternative Analysis**

In this data set the majority of participants (N=32) had SCR’s to more than 1/3 of the trials during acquisition. When excluding those participants (N=11) who had responded to less than 1/3 of trials in acquisition, the pattern of results does not change for SCR magnitude. We found a significant main effect of Stimulus, *F*(3,127.02) = 9.608, *p* < .001 and Phase, *F*(1,229.61) = 17.027 *p* < .001, as well as significant interaction between Stimulus x Phase, *F*(3,127.02) = 3.616, *p* = .015. Lastly, there was a significant interaction between Stimulus x Phase x IU, *F*(3,127.02) = 3.599, *p* = .015.

**Text D. Square root transformed SCR data without z-scoring**

The high response to GS2 during extinction at low (-1 SD) IU stands out and is puzzling, especially since we do not observe this when analyzing the square root transformed SCR data without z-scoring (see Fig A below). We think that this anomalous finding may be due to a number of factors: using a set random order for all participants, range correction, and the calculation of estimated marginal means, adjusting for variability in PSWQ and STAIX-2. Future research should control for these factors.

**
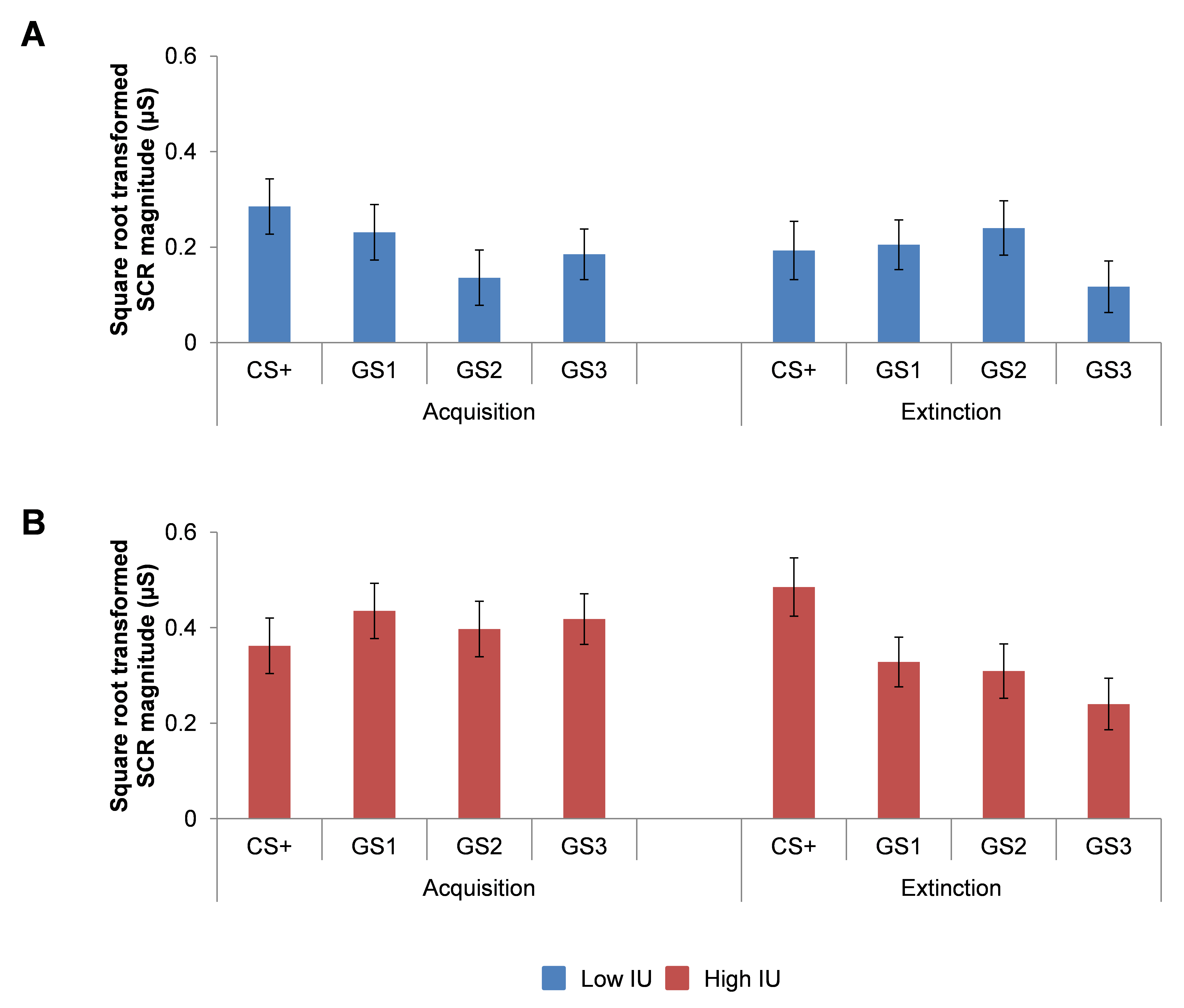
**

**Fig A***.* Bar graphs depicting IU estimated at + or - 1 SD of mean IU from the multilevel model analysis for SCR magnitude during acquisition, (A) and extinction, (B). Bars represent standard error at + or – 1 SD of mean IU. Square root transformed (μS), skin conductance magnitude measured in microSiemens.
